# Wētā Aotearoa—Polyphyly of the New Zealand Anostostomatidae (Insecta: Orthoptera)

**DOI:** 10.3390/insects15100787

**Published:** 2024-10-09

**Authors:** Steven A. Trewick, Briar L. Taylor-Smith, Mary Morgan-Richards

**Affiliations:** 1 Wildlife and Ecology, School of Food Technology and Natural Sciences, Massey University, Private Bag 11-222, Palmerston North 4442, New Zealand; m.morgan-richards@massey.ac.nz; 2 Ecology Group, Institute of Agriculture and Environment, Massey University, Private Bag 11-222, Palmerston North 4442, New Zealand; briar6@gmail.com

**Keywords:** king crickets, Stenopelmatoidea, mitogenomics, monophyly, systematics

## Abstract

**Simple Summary:**

Large crickets in the family Anostostomatidae, which are locally known as wētā, are a prominent feature of New Zealand endemic diversity and ecology. However, their systematics are poorly resolved, which reflects the wider uncertainty about the treatment on this orthopteran family. We examined the relationships among the New Zealand genera with representatives of the fauna from nearby Australia and New Caledonia by using a DNA dataset. We found that the New Zealand genera comprised four distinct lineages that were each more closely related to taxa outside New Zealand. We also found that the most speciose genus in New Zealand comprised two paraphyletic taxa.

**Abstract:**

The Anostostomatidae of Aotearoa New Zealand are well-characterized at the genus and species level, but the higher-level systematics of the family as a whole remain poorly resolved. We tested the hypothesis that the New Zealand anaostostomatid fauna consists of a single monophyletic group consistent with a single common ancestor. For phylogenetic analysis, we sampled the genera in Aotearoa New Zealand as well as representatives of the family from Australia and New Caledonia. Maximum likelihood analyses including topological comparison statistics with a DNA alignment of thirteen mitochondrial and four nuclear protein coding genes rejected the monophyly of lineages in New Zealand. We found phylogenetic support for four separate New Zealand lineages; three with their closest relatives in Australia and one in New Caledonia. The New Zealand genus *Hemiandrus* is paraphyletic and the establishment of a morphologically distinct genus is justified. We determined that six of the valid species previously placed in *Hemiandrus* form a distinct clade that we designated here as *Anderus* gen. nov. The putative *Hemiandrus* that we sampled from Australia was sister to neither of the New Zealand lineages.

## 1. Introduction

The Anostostomatidae (Orthoptera) comprises taxa with a predominantly Southern Hemisphere distribution [1], and about 302 valid species [2]. The majority of species are flightless nocturnal predators or scavengers that hunt on the ground and burrow in soil to conceal themselves during the day. The family is richly represented in Central and South Africa, Australasia, Central and South America, and is also found in North Africa through Asia including the Himalaya, India, and China. Species of Anostostomatidae are also known in Sri Lanka and on islands near Taiwan and south of Japan. Despite considering many genera of this family insufficiently studied, Gorochov (2021) [3] suggested two subfamilies with Anostostomatinae divided into nine tribes, and Lezininae not divided. The placement of anostostomatid genera within tribes must be considered preliminary, and additional phylogenetic studies are required to understand the deeper evolutionary relationships within this widespread family.

**Figure 1:**
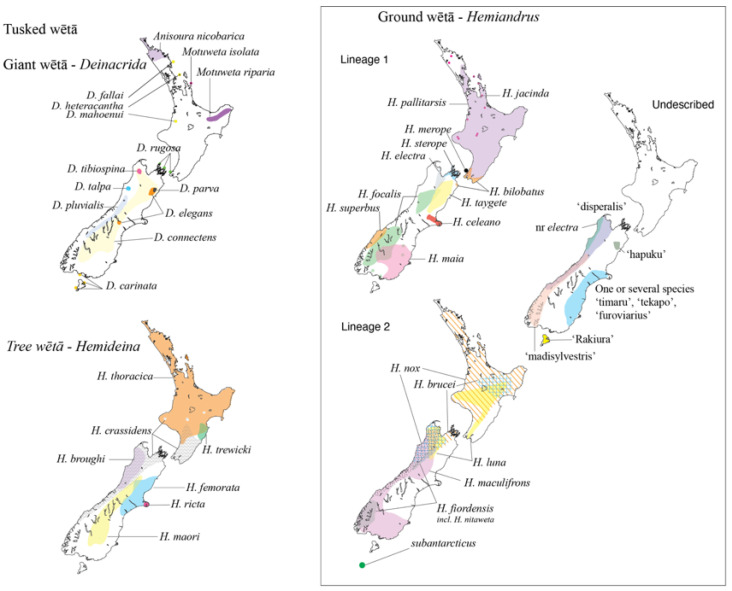
Diversity and approximate species ranges of wētā (Orthoptera: Anostostomatidae) in Aotearoa New Zealand.

In Aotearoa New Zealand, the family is represented by >40 species (Figure 1) in four informal groups of wētā: tree (*Hemideina* Walker), giant (*Deinacrida* White), ground (*Hemiandrus* Ander), and tusked (*Anisoura* Ander, *Motuweta* Johns). All are wingless. *Hemideina* and *Deinacrida* comprise most of the species of the small subfamily Deinacridinae [1], or all of the tribe Deinacridini [3], which are distinguished by stridulatory ridges, the musculature of their hind femura, and their predominantly herbivorous diet. These two genera are closely allied, and finding support for the reciprocal monophyly of the species within each of them has proven difficult [4,5,6,7]. Tree and giant wētā will scavenge invertebrate food but they are distinctive among anostostomatids in that they are primarily arboreal herbivores that feed on the foliage, flowers, and fruit of trees and shrubs [8,9]. This contrasts with, for example, the anostostostomatid fauna of Australia (king crickets), most of which forage on the forest floor, eating decaying material or are predatory [10].

**Figure 2:**
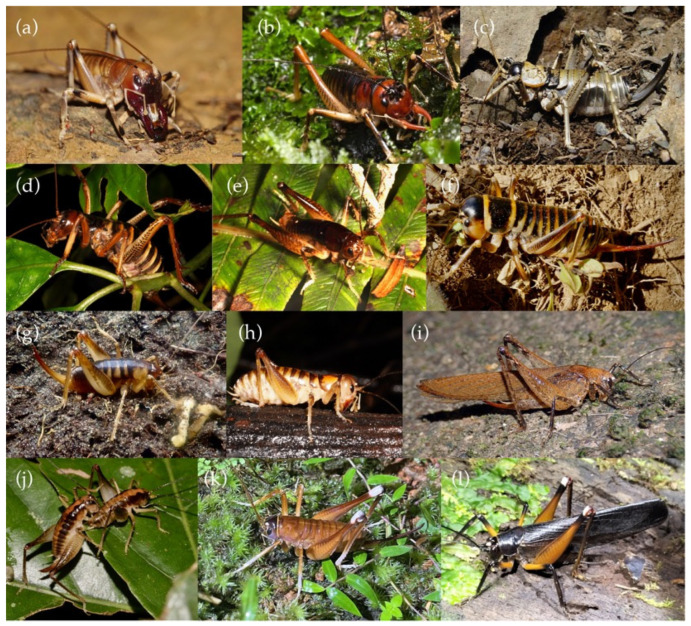
Species of Anostostomatidae included in this phylogenetic analysis. (**a**) *Carcinopsis* sp. male; (**b**) *Motuweta riparia* male; (**c**) *Deinacrida connectens* female; (**d**) *Hemideina crassidens* female; (**e**) *Hemiandrus brucei* male; (**f**) *Hemiandrus focalis* female; (**g**) *Hemiandrus* ‘rakiura’ female; (**h**) *Hemiandrus pallitarsis* female; (**i**) *Transaevum laudatum* female; (**j**) Australian ‘Hemiandrus’ sp. pair; (**k**) *Penalva flavoclceata* female; (**l**) *Exogryllacris ornata*, not to scale.

The New Zealand ground wētā (*Hemiandrus*) and tusked wētā (*Anisoura*, *Motuweta*) have a predominantly predatory diet and burrowing habit (with one exception) typical of Anostostomatidae elsewhere [11,12] (Figure 2). *Hemiandrus* and *Motuweta* were placed by Johns (1997) in the tribe Anostostomatini, along with 13 other genera from Australia, southern Africa, Madagascar, and South America [1]. Reappraisal a few years later left *Motuweta* in Anostostomatini, with just seven genera from Australia, New Caledonia, and southern Africa [3,13,14]. The tribal affinity of several genera was considered unclear [13], and the taxonomic uncertainty remains with *Hemiandrus* and *Carcinopsis*, among others in the subfamily Anostostomatinae but not assigned to a tribe [2]. Phylogenetic analyses based on short DNA sequences suggest the New Zealand tusked wētā (*Anisoura*, *Motuweta*) are monophyletic but not closely related to the New Zealand *Hemiandrus* [15,16]. The three tusked wētā species are united by the presence in adult males of prominent curved tusks protruding from the mandibles (Figure 2b) [17,18]. Salmon (1950) [19] placed the smallest (~20 mm long), and at that time only species, with the ground wētā as *Hemiandrus monstrosus*. However, unlike New Zealand *Hemiandrus*, the tusked wētā have auditory pits (tympana) on their fore tibiae. Johns (1997) [1] revised the name of the small tusked wētā, recognizing the precedence of *Anisoura nicobarica* Ander 1932 and placed it in subfamily Deinacridinae (i.e., with the tree and giant wētā). The name of this species appears to be the result of the mislabeling of the type specimens, as it does not occur in the Nicobar Islands in the Bay of Bengal.

In New Zealand, there are nineteen valid species of *Hemiandrus* and about six undescribed species in the grey literature referred to by unofficial tag-names [20,21]. In addition, there are undescribed species in Australia that may belong in the genus *Hemiandrus* [1,15]. Evolutionary relationships among the New Zealand anostostomatid fauna remain unclear, although, in agreement with the current taxonomy with tribal divisions [3], there is evidence that they are not a monophyletic group with respect to taxa outside New Zealand [15,22]. Although previous analyses involved short DNA sequences [5,6,15], these data and morphological examination [21] suggest polyphyly involving lineages in Australia and New Caledonia. Here, we specifically tested the hypothesis that the flightless wētā Aotearoa is a monophyletic group. This includes the possibility that *Hemiandrus* Ander (ground wētā), as currently applied, comprises two distinct lineages consistent with the resurrection of *Zealandosandrus* Salmon. We analyzed mitochondrial DNA genomes and nuclear genes from specimens representing the three prominent groups of New Zealand wētā with examples of Anostostomatidae from further afield to resolve these relationships. The current taxonomy is unclear about the tribal affiliations of *Hemiandrus*, so we included representatives of Anabropsini from Australia and Anostostomatini from New Caledonia as well as a species that represented the Australian putative *Hemiandrus* lineage. Additional sampling of *Hemiandrus* diversity in New Zealand was used for morphological examination and COI mtDNA sequencing to clarify membership to the two distinct clades we identified in the course of this work.

## 2. Materials and Methods

We sampled representatives of Anostostomatidae from Australia, New Zealand, and New Caledonia, seeking to assess the monophyly of the endemic New Zealand taxa (Table 1, Figure 1 and Figure 2). We included representation of New Zealand tusked (*Motuweta*), tree (*Hemideina*), giant (*Deinacrida*), and ground (*Hemiandrus*) wētā.

We used a skim-sequencing high-throughput next-generation sequencing approach [32,33,34] to generate DNA data for our samples, targeting the whole mitochondrial genome and nuclear histones. Insect DNA was extracted using a high salt method [6,35] and quantified using Qubit fluorometry (Life Technologies, Thermo Fisher Scientific Inc., Waltham, MA, USA). Genomic DNA samples were paired-end sequenced with high-throughput sequencing on an Illumina HiSeq 2500 (either BGI Genomics, Tai Po, Hong Kong or Macrogen Inc., Seoul, Republic of Korea) following fragmentation and indexing using the Illumina TruSeq Nano DNA Kit. The resulting 100 or 150 bp paired-end reads were filtered and edited to remove the sample barcodes and assembled in Geneious v9.1.4 [36].

Mitochondrial genomes were obtained from each specimen using an iterative reference mapping approach, starting with available short sequence data. Paired reads were iteratively mapped to the reference sequence in Geneious, generating a novel consensus sequence that was then used as a reference to remap the raw sequence reads. This process was repeated until all alignment gaps were filled by extension with the new sequence data and ambiguities resolved. Subsequent assemblies began with the more similar reference templates from our first anostostomatid mtDNA genomes. This approach has been proven to be fast and efficient for other Orthoptera [32,34,37]. Mitochondrial assemblies were uploaded as raw FASTA files for protein coding regions, rDNAs and tRNAs were identified using MITOS [38] for a comparison with the published data and detailed examination of the amino acid translations. Annotations were transferred and individually cross-checked through a comparison of the reading frames, amino acid translation, and RNA structure. A similar approach was used to assemble histone sequences using available Ensifera histone sequence and iterative assembly to extend across four exons (H2A, H2B, H3, and H4).

DNA sequence alignments were analyzed using maximum likelihood (ML) implemented in IQ-Tree v2.2 through IQ-Tree tools [39,40] utilizing model selection [41]) and ultrafast bootstrapping [42]. Partition models [43] were applied in ML analyses. Initially, gene and codon partitions were applied but these were optimized in IQTree v2.2 using the partition model test and merge functions to reduce overparameterization. A third codon RY coding scheme was also assessed using binary coding (0,1). Final trees used the optimal model scheme and 1000 bootstrap replicates. We repeated analyses using an unrooted tree approach to further assess the reciprocal monophyly of Australian and New Zealand anostostomatids in our sample.

We used tree constraints to test support for alternative topologies and making statistical comparisons of the fit of the data to the resulting ML topologies using the bootstrap proportion RELL (bp-RELL) approximation [44], (KH) Kishino–Hasegawa test [45], (SH) Shimodaira–Hasegawa test [46], (c-ELW) expected likelihood weights [47], and (AU) approximately unbiased test [48].

Topology constraint tests in Newick format:

**Hypothesis** **1.** Geographic monophyly but considering the *Motuweta/Carcinopsis* lineage as sister to core Anostostomatidae, as implied by preliminary analysis [15] *((Carcinopsis, Motuweta), (((‘Rakiura’, H. brucei, H. focalis, H. pallitarsis), (H. crassidens, D. connectens)), (E. ornata, ‘Hemiandrus’, P. flavocalceata, T. laudatum))).*

**Hypothesis** **2.** Geographic monophyly *(Carcinopsis, ((Motuweta, (‘Rakiura’, H. brucei, H. focalis, H. pallitarsis)), (H. crassidens, D. connectens)), (E. ornata, ‘Hemiandrus’, P. flavocalceata, T. laudatum)).*

**Hypothesis** **3.** Unconstrained tree *(Carcinopsis, Motuweta, ((((‘Rakiura’, H.brucei), (‘Hemiandrus’, T. laudatum)), ((P. flavocalceata, E. ornata), (H. focalis, H. pallitarsis))), (H. crassidens, D. connectens)).*

We identified two clades within our sampling of New Zealand *Hemiandrus* species, so we collected morphological information by examining additional species (Table 2). Thirteen species of *Hemiandrus* were sampled to encompass the known variation in traits (Table 2) [1,26,49,50,51]. Specimens were examined and anatomical features photographed using a SZX7 Zoom Stereomicroscope with SC100 digital camera and Cellsens v4.2 software from Olympus Corp., Tokyo, Japan. Amplification and sequencing were carried out using primers for mtDNA cytochrome oxidase subunit I (COI) C1-J–2195 [52] and mtd12_wetaR [53]. Previously published sequences [15,54] were obtained from GenBank. Sanger sequences were aligned and analyzed in Ugene [55].

## 3. Results

Seventeen protein coding genes comprising thirteen mtDNA and four nuclear histones were extracted from data for each of twelve species representatives, then concatenated, aligned, and trimmed, giving an alignment of 12,612 bp of DNA sequence, which formed the basis of the primary analysis (Table 1).

**Figure 3:**
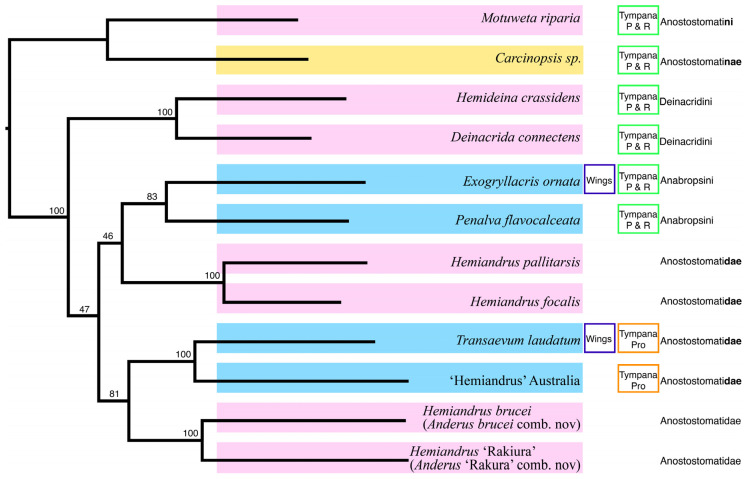
Maximum likelihood topologies inferred from 17 protein coding genes for 12 Anostostomatidae from New Zealand (pink), Australia (blue), and New Caledonia (yellow). Node support above 60% from 10,000 ML bootstrap replicates is shown. Two traits (presence/absence of tympana on the foretibia (P = prolateral; R = retrolateral), presence/absence of wings), and current higher-level classification [2] (Cigliano et al. 2024) are indicated.

Phylogenetic relationships were inferred from the combined mitochondrial and nuclear genes. A consistent topology was returned regardless of the partition models used including AA, 3rd codon exclusion from CDS, and RY (0,1) coding the 3rd codon position. Only the placement of the Deinacridini lineage (represented by *Deinacrida connectens* and *Hemideina crassidens*) varied, which is consistent with the short internal branch returned from the analyses (Figure 3). In all instances, the lineage comprising the New Zealand tusked wētā *Motuweta riparia* and the New Caledonian *Carcinopsis* was sister to the other sampled Anostostomatidae. This effectively resulted in the position of this lineage as the outgroup to the ingroup comprising four Australian and six other New Zealand taxa.

Among the Australian taxa in this set were two fully-winged species (Figure 2i,l), but they were not each other’s closest relatives. Instead, the two Australian representatives of the tribe Anabropsini were sister to one another, and the wingless Australian ‘Hemiandrus’ sp. was sister to the winged species *Transaevum laudatum* in all analyses (Figure 3).

**Figure 4:**
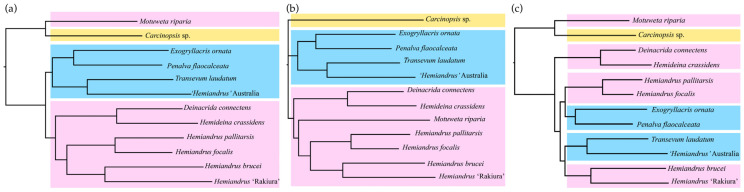
Maximum likelihood topologies inferred from 17 protein coding genes for 12 Anostostomatidae from New Zealand (pink), Australia (blue), and New Caledonia (yellow) with constraints: (**a**) hypothesis 1; (**b**) hypothesis 2; (**c**) hypothesis 3 (no constraint).

We assessed the possibility that the monophyly of the New Zealand taxa was statistically as likely as this by constraining the tree topology to enforce monophyly (Figure 4) and used 1000 RELL replicates. These analyses confirmed the best fit of the molecular data to the unconstrained phylogeny (Figure 4c, Table 3).

**Figure 5:**
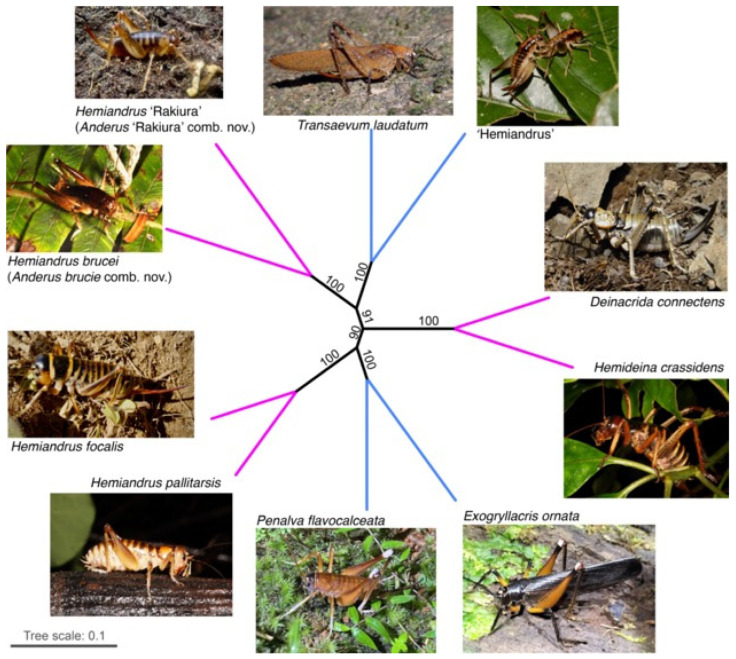
Evolutionary relationship of 10 Anostostomatidae species from Australia (blue) and New Zealand (pink) inferred with unrooted ML phylogeny with 1000 bootstrap replicates for DNA sequences from 13 mtDNA and 4 nuclear protein coding genes (12,612 bp).

We then removed the *Motuweta* and *Carcinopis* sister lineage and repeated the ML analysis with optimal partition models and found improved support for internal nodes in the resulting trees (Figure 5). Without other information, the root could fall on any edge of this unrooted topology (Figure 5), but all possible placements of the root nevertheless resulted in paraphyly of the Australian and New Zealand taxa in the analysis. Furthermore, no placement of a root resulted in the monophyly of Australian and New Zealand *Hemiandrus* or the monophyly of *Hemiandrus* in New Zealand, which comprises two independent lineages.

**Figure 6:**
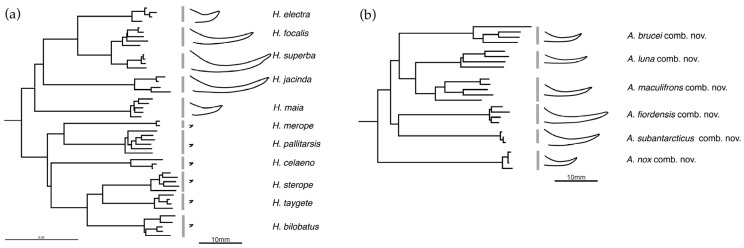
Neighbor-joining trees of available mitochondrial DNA COI (720bp) sequences for recognized species of New Zealand ground wētā that are currently placed in the genus *Hemiandrus*. The two lineages resolved as separate clades by the analysis of 17 protein coding genes (Figure 3) are presented here as (**a**) *Hemiandrus* Ander and (**b**) *Anderus* gen. nov. Silhouettes represent the approximate shape and size of adult female ovipositor profile of each species (10 mm scale bar). GenBank accession numbers in Table 2.

Having identified compelling phylogenetic evidence that the New Zealand representatives of the genus *Hemiandrus* were not monophyletic, we extended our sampling to determine which of the 19 valid species were part of the *Hemiandrus* clade and which were not. Using mitochondrial COI sequences, we identified seven species within the genus *Hemiandrus* and six species that formed a separate clade (Table 2, Figure 6). We propose a new genus to align with these evolutionary relationships.

### Taxonomy

Class Insecta
Order Orthoptera
Suborder Ensifera
Superfamily Stenopelmatoidea
Family Anostostomatidae Saussure (1859)
*Anderus* gen. nov.
zoobank.org:pub:65583178-0930-47F3-A9A8-C887572BC0A8

**Figure 7:**
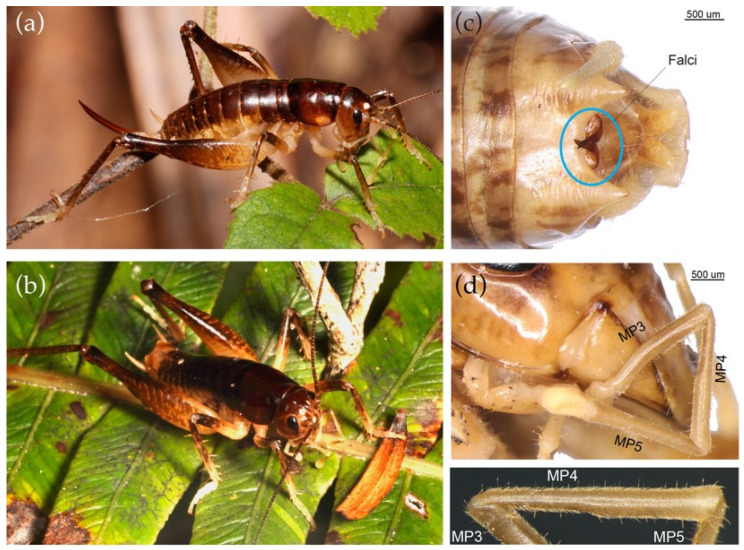
*Anderus brucei* comb. nov. (**a**) Adult female; (**b**) Adult male; (**c**) Dorsal male terminalia showing long falci in contact that is typical of *Anderus* gen. nov.; (**d**) Maxillary palp in situ and in silhouette showing the distribution of fine hairs along the full length of MP4, which is typical of *Anderus* gen. nov. (a, photocredit Uwe Schneehagen). Full description of *Anderus brucei* comb. nov. is available in Taylor-Smith et al. (2016) [26]. Falci are, as defined by Johns 2001, are the sclerotized projections on the tenth tergite of males [20]. Although the etymology was not given, the term most probably derives from the sickle-like shape of the structures in many taxa (Falx in Latin).

Nocturnal anostostomatids lacking wings. Small to medium (body length approximately 8 to 15 mm) in size and pigmentation varied. Males and females similar in size or females slightly larger. Leg, head, and mandible dimensions are similar in the two sexes. All have maxillary palps with dense small hairs extending through the 5th, 4th, and 3rd segments (c.f. *Hemiandrus* in which pilosity is on the 5th and distal half of the 4th segments only). Some longer setae scattered on the 3rd and 4th segments. Adult females of all species have prominent, long, narrow curved, or almost straight ovipositors. Adult males have a pair of relatively long and straight, pointed falci on the 10th tergite meeting or overlapping (c.f. *Hemiandrus* typically with short, hooked, or knob-shaped falci) (Figure 7). Foretibia lack tympana.

Designated type species: *Anderus brucei* (Formerly *Hemiandrus brucei*: [26]) (Figure 7). *Anderus* gen. nov. also includes the species *A. fiordensis* [19] (NB: the species *H. nitaweta* Jewell is a synonym of this species); *A. maculifrons* [28]; *A. luna* [26]; *A. subantarticus* [19]; *A. nox* [26]. The undescribed species included in our main analysis (Figure 5) as ‘Rakiura’ from Stewart Island (=‘saxatilis’ [20]) also belongs to *Anderus* gen. nov. and awaits formal description.

## 4. Discussion

Recent subdivisions of the family Anostostomatidae into tribes based on morphological characters have left a number of genera unplaced due to the lack of suitable morphological evidence [1,2,3]. Two of these unclear genera, *Hemiandrus* and *Transaevum*, are represented in our phylogenetic analysis. Based on our sampling, we concluded that both genera are more closely related to representatives of the tribe Anabropsini than either Deinacridini or Anostostomatini. In the present analysis, we tested the hypothesis that the extant, flightless Anostostomatidae of Aotearoa New Zealand form a monophyletic group, and found that this could be rejected. Instead, we identified four lineages of wētā Aotearoa that are each more closely related to taxa in either Australia or New Caledonia than to other wētā Aotearoa.

**Figure 8:**
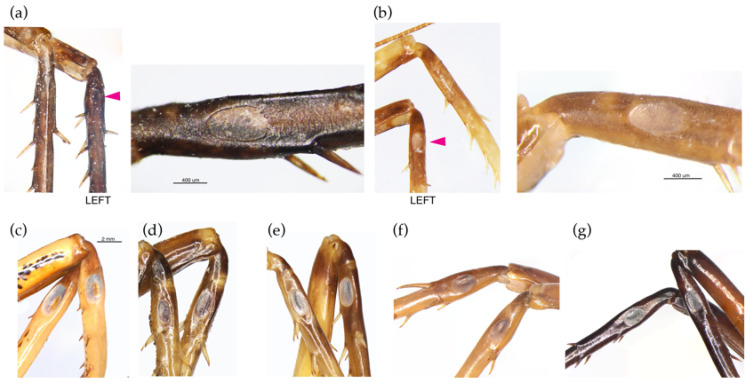
Anostostomatid tympana on fore tibiae. Views of paired legs provides a view of both pro- and retro-lateral surfaces: (**a**) *Transaevum laudatum* and (**b**) Australian ‘Hemiandrus’ have shallow prolateral tympana (pink arrows); (**c**) *Hemideina crassidens*; (**d**) *Motuweta riparia*; (**e**) *Carcinopsis* sp.; (**f**) *Penalva flavocalceata* and (**g**) *Exogryllacris ornata* have prominent paired prolateral and retrolateral tympana.

We found that the tusk wētā lineage (*Motuweta*) was more closely related to New Caledonian taxa, represented here by *Carcinopsis*, than to other New Zealand genera. Together, *Motuweta* and *Carcinopsis* form a lineage that appears to be sister to the core Anostostomatidae, at least as represented in the present sample. Although adult males of both these genera have exaggerated mandible structures that are probably associated with male–-male sexual competition, the structures involved differ in form [56]. In the New Zealand *Motuweta* (and *Anisoura*), each mandible is relatively small but bears a long, curved projection (tusk) whereas in males of *Carcinopsis* that have exaggerated structures, it is the mandibles themselves that are enlarged [23,57]. These are among the paraphyletic diversity of secondary sexual head structures displayed by male Anostostomatidae around the world [58]. Analysis including wider representation of global ansostostomatid diversity to resolve the deeper systematic relationships and evolution of these interesting traits requires a similar scale of DNA sequence data as presented here, as short DNA sequence data are not sufficient [15,16]. The New Zealand genus *Hemiandrus* emerges as paraphyletic, comprising two separate lineages of ‘ground’ wētā with independent ancestry among Australasian Anostostomatidae. The undescribed Australian species that were proposed as belonging to *Hemiandrus* [1] belong to another independent lineage that is not closely allied to either of the New Zealand *Hemiandrus* lineages. In the context of the present sampling, the undescribed Australian ‘Hemiandrus’ species is sister to the Australian winged *Transaevum laudatum.* It is notable that both taxa bear a single prolateral tympanum on each fore tibia (Figure 8), whereas none of the *Hemiandrus* in New Zealand have any tympana. The tympanum was noted in the description of *Transaevum* but not in Australian putative ‘Hemiandrus’ discussed in the same paper [1].

Ander (1838) [59] proposed the name *Hemiandrus* for relatively small New Zealand anostostomatids in which the females have minute ovipositors, making it difficult to distinguish the sexes (hence half male). Salmon (1950) [19] proposed the name *Zealandosandrus* for New Zealand ground wētā species where the females have a long ovipositor, and retained *Hemiandrus* for species where females have very short ovipositors. However, ovipositor length has emerged as a paraphyletic trait. Although one lineage of New Zealand ground wētā includes a distinctive radiation of species with minute ovipositors such as *H. bilobatus* Ander, 1938 [51], it also includes some species with long ovipositors such as *H. focalis* (Hutton) and *H. jacinda* Trewick as well as others with intermediate length ovipositors (*H. electra*, *H. maia* [49]) (Figure 6). Salmon (1950) [19] also confounded species of different lineages by confusing *H. focalis* with *H. maculifrons*, making the name *Zealandosandrus* unclear and unusable [1]. Thus, we propose the name *Anderus* gen. nov., masculine, in honor of Kjell Ernst Viktor Ander (1902–1992), a Swedish entomologist who contributed to the systematics of Ensifera and established the genus *Hemiandrus*. *Anderus* gen. nov. is readily distinguished from *Hemiandrus* by pilosity on the maxillary palps that that extends over all of MP4 and part of MP3 (Figure 7, Table 2) [20,21].

There remain many questions about the higher-level systematics of the family Anostostomatidae and related Orthoptera [16], and identifying the polyphyly of the living representatives of the family in Aotearoa New Zealand indicates that inferences from biogeography should not overly influence this work. Although not ubiquitous in the Australian Anostostomatidae fauna, several fully winged species exist, and partial or fully developed wings also occur in some Ansostostomatidae in Asia and the Americas (e.g., [14,60,61]). While none of the extant wētā of Aotearoa New Zealand have wings, it is now apparent that this endemic fauna is not derived from a single common ancestor in New Zealand. Given the abundant evidence of long-distance dispersal of Rhaphidophoridae in the region [37,62], there is no reason to exclude either active dispersal (flying) or passive dispersal (rafting) of Anostostomatidae to explain the ancestral relationships inferred here. This mirrors the situation in some birds, where island faunas often comprise high numbers of independently derived endemic flightless species (e.g., [63,64,65]). Most profoundly, the large moa (Dinornithiformes) that lacked all bones of the forelimb, which, prior to recent appropriate phylogenetic analysis, were presumed to have a flightless ancestor along with other members of the order. It is now clear that moa and other ‘ratite’ lineages most likely had flying ancestors with that critical trait lost after colonization of separated lands [66]. The absence, in extant species, of traits (such as wings) that are ecologically and taxonomically influential, challenges biogeographic interpretation [67,68], but reemphasizes the dynamism of evolutionary biology.

## 5. Conclusions

With a small number of representative taxa and rich multigene mitochondrial and nuclear DNA sequence data, we found a convincing signal of paraphyly among Anostostomatidae from New Zealand, Australia, and New Caledonia. In doing so, we found that the genus *Hemiandrus* actually comprises two separate genera that are both endemic to New Zealand. We propose a new genus (*Anderus* gen. nov) to accommodate this.

## Figures and Tables

**Table 1:** Anostostomatidae sampled for whole mitochondrial genomes and nuclear loci. Voucher material is in the Phoenix Collection at Massey University, Palmerston North (MPN). Current classification within Anostostomatidae, after Cigliano et al. (2024) [2]. * Undescribed but proposed as synonymous with New Zealand *Hemiandrus*. Images of vouchers for undescribed taxa can be found at: MPN_GW1392 https://inaturalist.nz/observations/9593056, MPN_ORT190 https://inaturalist.nz/observations/223875743.

Taxon	Classification	Location	MPN Code	GenBank mtDNA	GenBank Histone	Collector
*Carcinopsis* sp. Brunner 1888 [23]	Anostostomatinae	Col d’Amieu, New Caledonia	ORT388	PQ442192	PP965128, PP965140, PP965152, PP965164	E.X.M. Trewick
*Deinacrida connectens* (Ander, 1939) [24]	Deinacridini	Mount Peel, South Island, New Zealand	Dco2011	PQ442198	PP965131, PP965143, PP965155, PP965167	S.A. Trewick
*Exogryllacris ornata* Willemse, 1963 [25]	Anabropsini	Bartle Frere, Queensland, Australia	ORT193	PQ442190	PP965130, PP965142, PP965154, PP965166	G. Monteith
*Hemiandrus* ‘Rakiura’	Unplaced	Tin Range, Rakiura, New Zealand	GW1392	PQ442194	PP965139, PP965151, PP965163, PP965175	D. Hegg
*Hemiandrus brucei* Taylor-Smith, 2016 [26]	Unplaced	Whanganui, North Island, New Zealand	Hbr2011	PQ442197	PP965138, PP965150, PP965162, PP965174	B.L. Taylor-Smith
*Hemiandrus focalis* (Hutton, 1897) [27]	Unplaced	Lake Alta, South Island, New Zealand	GW262	PQ442196	PP965134, PP965146, PP965158, PP965170	M. Morgan-Richards
*Hemiandrus pallitarsis* (Walker, 1869) [28]	Unplaced	Palmerston North, North Island, New Zealand	Hpa2012	PQ442195	PP965133, PP965145, PP965157, PP965169	S.A. Trewick
‘Hemiandrus’ sp. [1]	Unplaced	Bellenden Ker, Queensland, Australia	ORT190	PQ442193	PP965137, PP965149, PP965161, PP965173	G. Monteith
*Hemideina crassidens* (Blanchard, 1851) [29]	Deinacridini	Maitai Valley, South Island, New Zealand	Hcr2011	PQ452770	PP965132, PP965144, PP965156, PP965168	S.A. Trewick
*Motuweta riparia* (Gibbs, 2002) [30]	Anostostomatini	Raukumura Range, North Island, New Zealand	TW29	PQ423746	PP965129, PP965141, PP965153, PP965165	E. Dowle
*Penalva flavocalceata* (Karny, 1929) [31]	Anabropsini	Bartle Frere, Queensland, Australia	ORT200	PQ442191	PP965136, PP965148, PP965160, PP965172	G. Monteith
*Transaevum laudatum* (Johns, 1997) [1]	Unplaced	Mt Finnigan, Queensland, Australia	ORT178	PQ442189	PP965135, PP965147, PP965159, PP965171	G. Monteith

**Table 2:** Aotearoa New Zealand ground wētā (*Hemiandrus* and *Anderus* gen. nov.) used for morphological and mtDNA COI examination.

Species	MPN Code	Location	Year	GenBank Accession
*Hemiandrus brucei* (*Anderus brucei* nov. comb.)	GW218	Raurimu	2007	EU676796
	GW126	Pureora Forest	2005	EU676793
	GW49A	Puketi Forest	1990	EU676765
	GW04	Manganuku	1998	EU676798
	GW93A	Pelorus Bridge	2005	EU676791
*Hemiandrus luna* (*Anderus luna* nov. comb.)	GW104	Sky Farm	2006	EU676742
	GW143	Lewis Pass	2006	EU676784
	GW1385	Tongariro	2021	PP34546
	GW916B	Arthurs Pass	2013	PP34545
*Hemiandrus fiordensis* (*Anderus fiordensis* nov. comb.)	GW1481	Stuart Mountains	2022	PP34550
	FD3(*nitaweta*)	Sinbad Valley	2013	PP34541
	GW70	Lake Roe	2004	PP34547
	GW1516	Shy Lake	2022	PP34553
*Hemiandrus nox* (*Anderus nox* nov. comb.)	GW834	Hokitika	2012	PP34552
	GW76	Awakiri Valley	1997	EU676766
	GW896a	Denniston Plateau	2012	PP34549
	GW899	Denniston Plateau	2013	PP34548
	GW1542	St Arnaud	2005	PP34551
*Hemiandrus maculifrons* (*Anderus maculifrons* nov. comb.)	GW119	Catlins Coast	2006	EU676770
	GW201	Takitimu Mountains	2006	EU676772
	GW68	Mount Fyffe	2004	EU676787
	GW217	Kahurangi	2007	EU676776
	GW150	Franz Josef	2006	EU676786
*Hemiandrus subantarcticus* (*Anderus subantarcticus* nov. comb.)	GW988	The Snares	2010	MW463359
	GW792	The Snares	2010	PP34544
	GW989	The Snares	2010	MW463360
*Hemiandrus electra*	GW138	St Arnaud	2005	EU676783
	GW1028	Mount Richmond	2013	PP34562
	GW1029	Mount Richmond	2013	PP34561
*Hemiandrus focalis*	GW1212	Takitimu Mountains	2019	PP34554
	GW1211	Takitimu Mountains	2019	PP34555
	FD7	Eyre Mountains	1999	PP34542
	GW206	Obelisk	2006	EU676774
	GW08	Harris Saddle	1999	EU676773
*Hemiandrus superbus*	FD5	Sinbad Valley	2013	PP34543
	FD10	Sinbad Valley	2010	MW463358
	FD8	Sinbad Valley	2011	MW463357
	GW1198	Skippers Range	2019	PP34556
*Hemiandrus jacinda*	GW1376	Pouakai	2021	PP34560
	GW1486	Pukeiti	2022	PP34559
	GW208	Whareorino	2006	MW463354
	GW1328	Thames	2020	MW463352
	GW62	Moehau	1990	MW463353
*Hemiandrus maia*	GW125	Portabello	2006	EU676744
	GW1067	Mount Kyeburn	2013	PP34557
	GW118	Hampden	2006	EU676795
	GW136	Blue Mountains	2006	EU676780
*Hemiandrus merope*	GW674	Kapiti Island	2011	MT6323126
	GW682	Kapiti Island	2011	MT623127
*Hemiandrus palllitarsis*	GW227	Hauturu	2007	JF895541
	GW226	Hauturu	2007	JF895542
	KA90	Kauaeranga Valley	2012	JF895543
	KA88	Kauaeranga Valley	2012	F895547
	MI98	Middle Island	2012	JF895548
	CO145	Moehau	2012	JF895551
	GW87	Pohaninga Valley	2004	JF895554
*Hemiandrus celaeno*	GW120	Banks Peninsular	2005	EU676771
	GW127	Foggy Peak	2006	EU676778
	GW129	Foggy Peak	2006	EU676779
*Hemiandrus sterope*	GW1033	Lewis Pass	2014	MT623110
	GW1047	Cable Bay	2014	MT623103
	GW717	Manaroa	2012	MT623102
	GW602	Te Rua Bay	2010	MT623101
	GW54	Whites Bay	1990	EU676788
*Hemiandrus taygete*	GW491	Kaikoura	2009	MT623112
	GW1031	Mount Richmond	2013	MT623115
	GW871	Upper Clarence Valley	2012	MT623113
	GW1231	Youngman Stream	2019	PP34558
*Hemiandrus bilobatus*	GW586	Awatere Valley	2010	MT623085
	GW25	Wellington	1999	EU676794
	GW240	Wellington	2007	JF895562
	GW657	Mana Island	2010	MT623095
	GW122	Marfells Beach	2000	EU676777
	GW55	Marfells Beach	1990	EU676789
	GW193	Muritai	2006	JF895564

**Table 3:** Support for alternative evolutionary topologies among Anostostomatidae. Comparison of trees that constrained the relationships based on geography (Hypotheses 1 and 2) or unconstrained (Hypothesis 3). Results of 1000 RELL replications for 12 Anostostomatidae comparing the ML fit of data (12,612 bp DNA sequence) to each of three hypotheses (see Figure 4).

Hypothesis	logL	deltaL	bp-RELL	p-KH	p-SH	c-ELW
1	−89,689.86177	679.86	0−	0−	0−	3.53 × 10^−231^−
2	−90,220.76741	1210.8	0−	0−	0−	0−
3	−89,010.00659	0	1+	1+	1+	1+

deltaL: logL difference from the maximal logl in the set. Bp-RELL: bootstrap proportion using RELL method [44]. P-KH: *p*-value of one-sided Kishino–Hasegawa test [45]. P-SH: *p*-value of Shimodaira–Hasegawa test [46]. C-ELW: expected likelihood weight [47]. Plus signs denote the 95% confidence sets. Minus signs denote significant exclusion.

## Data Availability

The original data presented in the study are openly available in the NCBI GenBank database https://www.ncbi.nlm.nih.gov/genbank/. See Table 1 and Table 2 for accession numbers.
